# A rare missense variant impacting NEK1 kinase function is associated with ALS

**DOI:** 10.1186/s40478-026-02351-6

**Published:** 2026-06-25

**Authors:** David Brenner, Anna Ponomarenko, Iris Petrut, Sofia Beyrle, Matilde Contardo, Isabel Loss, Constantin Radke, Jonas Frank, Eleni Zimmer, Matthias Schlesner, Pascal Achenbach, Wendy Scheveneels, Amr Aly, Hülya Nazlican, Jasper Hesebeck-Brinkmann, Patrick Oeckl, Kathrin Müller, Reiner Siebert, Tobias Böckers, Kristel van Eijk, Jan Veldink, Alexander Kleger, Medhanie Mulaw, Peter M. Andersen, Karin Forsberg, Jochen H. Weishaupt, Seyed Babak Loghmani, Thorsten Grehl, Philip van Damme, Joachim Weis, Alberto Catanese

**Affiliations:** 1 Department of Neurology, University Hospital Ulm, 89081 Ulm, Germany; 2 Center for Rare Diseases (ZSE) Ulm, Ulm University Hospital Center for Rare Diseases, 89081 Ulm, Germany; 3 German Center for Neurodegenerative Diseases (DZNE), Ulm site, 89081 Ulm, Germany; 4 Institute of Anatomy and Cell Biology, Ulm University School of Medicine, 89081 Ulm, Germany; 5 Department of Neurosciences, Laboratory of Neurobiology and Leuven Brain Institute (LBI), KU Leuven-University of Leuven, 3000 Leuven, Belgium; 6 VIB, Center for Brain & Disease Research, 3001 Leuven, Belgium; 7 Institute of Neuropathology, RWTH Aachen University Hospital, Pauwelsstrasse 30, 52074 Aachen, Germany; 8 Biomedical Informatics, Data Mining and Data Analytics, University of Augsburg, 86159 Augsburg, Germany; 9 Institute of Molecular Oncology and Stem Cell Biology (IMOS), Ulm University Hospital, 89081 Ulm, Germany; 10 Brain Science Institute, Johns Hopkins University School of Medicine, Baltimore, MD USA; 11 Department of Neurology, Johns Hopkins University School of Medicine, Baltimore, MD USA; 12 Department of Neurology, Centre for ALS and Other Motor Neuron Disorders, Alfried Krupp Krankenhaus Rüttenscheid, 45131 Essen, Germany; 13 Institute of Human Genetics, Ulm University and Ulm University Medical Center, Ulm, Germany; 14 Department of Neurology, Brain Centre Rudolf Magnus, University Medical Centre Utrecht, Utrecht University, 3584 CG Utrecht, The Netherlands; 15 Division of Interdisciplinary Pancreatology, Department of Internal Medicine I, Ulm University Hospital, 89081 Ulm, Germany; 16 Core Facility Organoids, Ulm University, 89081 Ulm, Germany; 17 Unit for Single-Cell Genomics, Medical Faculty, Ulm University, 89081 Ulm, Germany; 18 Department of Clinical Sciences, Neurosciences, Umeå University, Umeå, Sweden; 19 Department of Neurology, University Hospitals Leuven, 3000 Leuven, Belgium; 20 Institute of Neuroanatomy, University Clinic RWTH Aachen, Wendlingweg 2, 52074 Aachen, Germany

**Keywords:** NEK1, ALS, Genetics, Missense variant, Kinase, TDP-43 pathology, Autophagy

## Abstract

Heterozygous truncating loss-of-function (LoF) variants in *NEK1* are a known cause of amyotrophic lateral sclerosis (ALS). *NEK1* encodes the pleiotropic serine/threonine kinase NIMA-related kinase 1, and prior in vitro studies have implicated kinase dysfunction as the principal pathogenic mechanism underlying NEK1-associated ALS. However, bona fide pathogenic missense variants causally linked to ALS have not previously been reported, leaving this hypothesis unconfirmed. Here, we identify a rare NEK1 missense variant, p.N598S, that co-segregates with disease in a familial ALS pedigree and is enriched in European ALS cohorts. This variant exhibits normal protein expression levels, indicating a functional rather than quantitative defect. Using isogenic human motor neurons, we directly compared the effects of p.N598S with those of the ALS-associated truncating variant p.R812* to delineate disease mechanisms. The p.N598S variant induced pathological phenotypes consistent with NEK1 haploinsufficiency, including increased susceptibility to DNA damage, increased apoptosis, ciliary dysmorphia, and nucleocytoplasmic translocation of TDP-43. Importantly, p.N598S impaired NEK1 kinase activity, and pharmacological inhibition of NEK1 recapitulated the cellular phenotypes observed in both p.N598S- and p.R812*-mutant motor neurons. Collectively, these findings provide strong genetic and functional evidence for a disease-causing role of NEK1 kinase disruption in NEK1-ALS. Our findings provide immediate diagnostic and therapeutic implications, particularly for the functional interpretation of missense variants of uncertain significance and the development of targeted treatment strategies.

**Supplementary Information:**

The online version contains supplementary material available at 10.1186/s40478-026-02351-6.

## Introduction

Amyotrophic lateral sclerosis (ALS) is a fast-progressive neurodegenerative disease characterized by the loss of motor neurons in cortex, brainstem and spinal cord which leads to death within a few years after symptom onset. While familial ALS, defined by a positive family history, accounts for 5–10% of cases, approximately 13% of individuals with sporadic ALS (with no known family history) also carry pathogenic genetic variants [1–3]. Heterozygous nonsense loss-of-function (LoF) variants in the *NEK1* gene are responsible for 2% of familial and sporadic ALS cases [4, 5]. *NEK1* encodes NIMA-related kinase 1, an enzyme crucial for several cellular processes, including DNA damage checkpoint control, DNA damage repair, cell cycle control, and ciliogenesis [6, 7]. Missense variants (including the single-nucleotide polymorphism p.R261H) occur in another 2–3% of ALS cases [2–5]. Although the p.R261H variant has been linked to an increased risk of ALS, it is not a direct cause of the disease [5]. Other, rarer missense variants have not been collectively found enriched in ALS, nor have any dominantly inherited familial ALS cases been linked to a missense variant in *NEK1* [4, 5]. Some missense variants may nevertheless be pathogenic if they disrupt a critical function of NEK1. Previous in vitro studies have shown that pharmacological inhibition of NEK1 kinase activity or introducing an engineered kinase-dead variant can replicate phenotypes seen in pathogenic truncating *NEK1* variants in vitro, such as axonal impairment, DNA damage accumulation, and ciliary dysfunction [8–10]. This suggests that the loss of kinase activity may be the primary disease mechanism in NEK1-ALS. However, until now, this hypothesis lacked in vivo confirmation in the form of identification of a kinase-dead missense variant associated with ALS.

Here, we report the identification of a rare missense variant in *NEK1* that maintains protein expression but disrupts NEK1 kinase function. This variant is sufficient to cause ALS in humans and induce phenotypes associated with NEK1 haploinsufficiency in human motor neuron cultures. Our findings provide the first in vivo confirmation that heterozygous disruption of NEK1 kinase function is the disease-defining event in NEK1-ALS, which has important implications for therapeutic strategies and the interpretation of missense variants of uncertain significance.

## Results

**Fig. 1:**
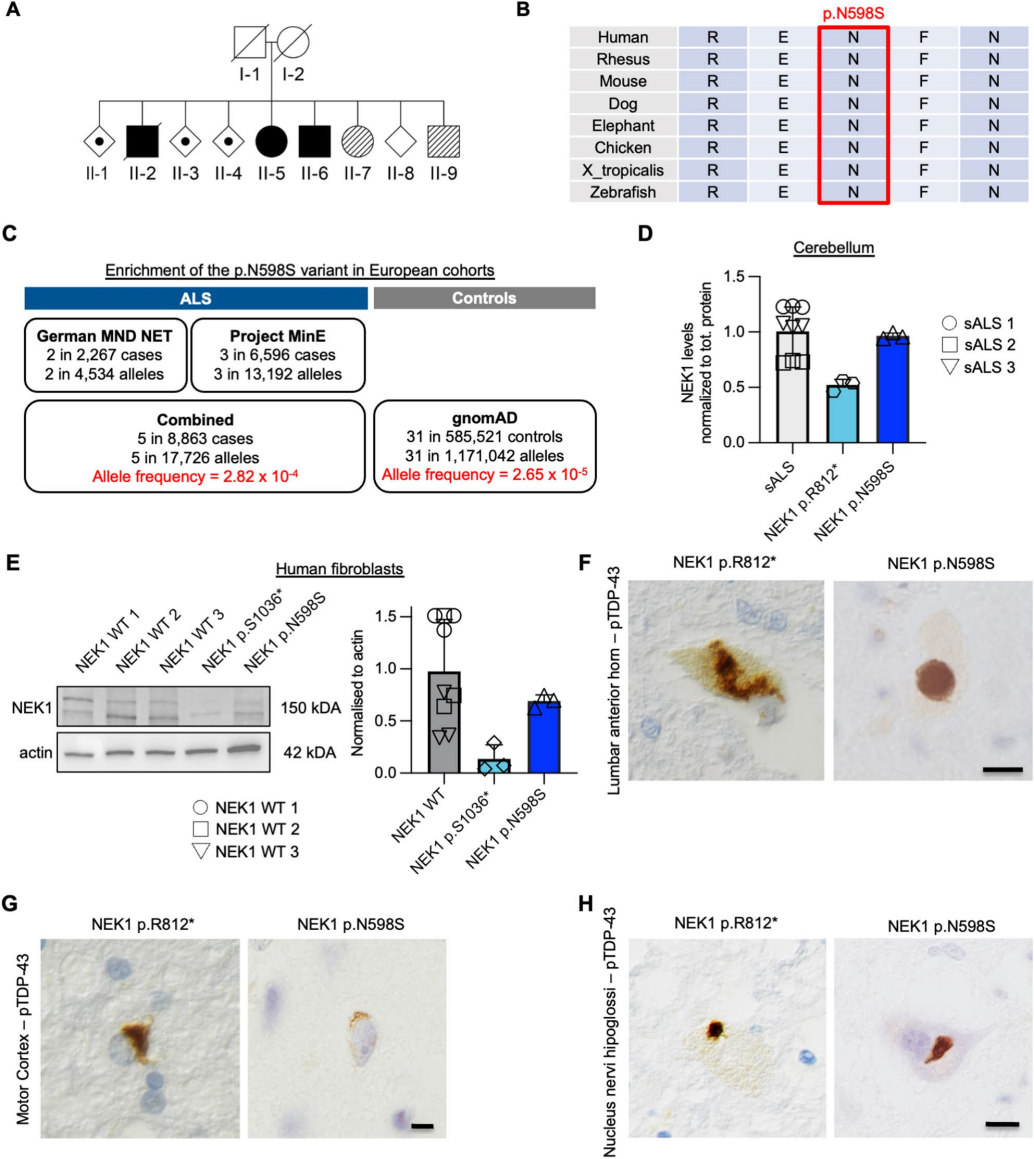
The rare NEK1 missense variant p.N598S causes familial amyotrophic lateral sclerosis.** A** Pedigree of ALS family carrying the NEK1 missense variant p.N598S. **B** Conservation of p.N598 (highlighted in red) across various species. **C** Burden analysis of the p.N598S variant in ALS patient cohorts of European ancestry (German MND NET and ProjectMinE) and reference database (gnomAD, only considering European cases). **D** ELISA against NEK1 in cerebellar autopsy lysates from ALS patients carrying p.N598S or p.R812* and sporadic ALS patients (3 technical replicates for each patient and control). **E** Western Blot against NEK1 in lysated fibroblasts cultured from skin biopsies of ALS patients carrying p.N598S or the truncating p.S1036* variant, and age-matched control probands (3 technical replicates for each patient and control).** F**–**H** pTDP-43 immunohistochemistry (DAB) shows ALS-typical nucleocytoplasmic translocation in cortical, bulbar and spinal motor neurons of p.N598S- and p.R812*-mutant ALS patients

The advent of tofersen, a treatment for SOD1-associated amyotrophic lateral sclerosis (ALS), and the discovery that a relevant proportion of sporadic ALS cases are caused by pathogenic genetic variants have made genetic testing a crucial component of ALS diagnosis [2, 3]. During routine genetic panel testing, we identified a rare, novel missense variant of uncertain significance in the *NEK1* gene in a patient with a positive family history of ALS (patient II.5 in Fig. 1A): NM_001199397.3(NEK1):c.1793 A > G (p.Asn598Ser). This variant, designated p.N598S, was the only variant found in this patient among a comprehensive list of known ALS-associated genes (see Methods). According to the GnomAD database, N598S is very rare in the general population, with a minor allele frequency (MAF) of 2.65 × 10^−5^ in European control populations. The amino acid residue at this position is highly conserved across multiple species (Fig. 1B; Supplementary Fig. 1). In silico pathogenicity prediction tools provided mixed results regarding the variant’s potential to cause disease. SIFT predicted the variant to be deleterious with low confidence, while PolyPhen classified it as “possibly damaging”. The CADD score for p.N598S was 24.8, suggesting that it is potentially harmful. Of note, two cases of the severe ciliopathy “short-rib thoracic dysplasia 6 with or without polydactyly” (OMIM phenotype number # 263520), caused by bi-allelic pathogenic variants in *NEK1*, carrying p.N598S variants have been submitted to ClinVar (ClinVar variation ID: 348110), suggesting that p.N598S disrupts a fundamental function of NEK1. Taken together, these findings supported the need for further functional investigation into this novel *NEK1* variant.

Genotyping confirmed that four other family members with confirmed or suspected ALS also carried the p.N598S variant (Fig. 1A, patients II.2, II.6, II.7, and II.9). Further genetic panel testing ruled out pathogenic variants in other known ALS genes for patients II.2 and II.6. The p.N598S variant was also identified in additional four patients with ALS from the German MND-NET and Project MinE cohorts [2, 5]. The clinical characteristics and disease courses of all p.N598S-positive patients are summarised in Tables 1 and 2 and described in more detail in the Methods section. In total, the p.N598S variant was identified in eight patients with confirmed ALS, five of whom were apparently unrelated. While kinship analysis was only feasible for the three Project MinE samples (Table 1, patients #7–9), it confirmed that these patients were not related. Comparing the p.N598S allele frequency in European ALS cohorts with that in a European control population from the gnomAD v4 database revealed a significant enrichment of this variant in ALS cases (odds ratio [OR] = 10.7, 95% confidence interval [CI] 4.15–27.42; *p* = 0.00019, Fisher’s exact test; Fig. 1C). Specifically, the allele frequency was 2.82 × 10^− 4^ in ALS cases (five out of 17,726 alleles), which is more than ten times higher than in the control group, where the frequency was 2.65 × 10^− 5^ (31 out of 1,171,042 alleles).

**Table 1:** Clinical measures of ALS patients bearing the p.N598S variant

Case nr.	Family history	Pedigree ID	Nationality	Sex	Site of onset	Age of onset	MND phenotype	ALSRS-*R* slope (points per month)	Survival (time to death or last follow-up), years	Status	Last ECAS	Smoker	Cormorbidity	Method of genetic testing
#1	pos	II.2	Ger	m	spinal (hand)	62	Classical ALS	3.33	1.5	dead	58	yes		Panel
#2	pos	II.5	Ger	f	spinal (hand)	55	Classical ALS	0.34	> 5	alive	102	yes	COPD, Membranous glomerulonephritis	Panel
#3	pos	II.6	Ger	m	spinal (leg)	53	UMN-dominant ALS	0.19	> 6.5	alive	41	yes	Suspected primary progressive multiple sclerosis	WES
#4	pos	II.7	Ger	f	spinal	56	Suspected ALS	NA	> 0.5	alive	NA	yes		PCR
#5	pos	II.9	Ger	m	spinal	50	Suspected ALS	NA	> 0.5	alive	NA	yes	COPD	PCR
#6	neg	–	Ger	f	bulbar	80	Classical ALS	3.1	0.91	dead	NA	no		Panel
#7	neg	–	Dutch	m	spinal (hand)	72	Segmental spinal muscular atrophy	NA	> 12	alive	NA	NA		WGS
#8	neg	–	Dutch	m	spinal	60	NA	NA	2.13	dead	NA	NA		WGS
#9	neg	–	US	f	spinal	63	NA	NA	NA	NA	NA	NA		WGS

#1–6: German MND NET; #7–9: ProjectMINE cohort

**Table 2:** Alignment with diagnostic criteria for ALS

Case nr.	Familiy ID	Revised El Escorial	Awaji-shima	Gold coast	Key diagnostic features
#1	II.2	Definite ALS	Definite ALS	ALS	UMN + LMN in bulbar, cervical, lumbosacral; rapid progression
#2	II.5	Definite ALS	Definite ALS	ALS	Multiregional UMN + LMN; slow progression
#3	II.6	Possible ALS	Possible ALS	ALS	UMN-dominant; no LMN/EMG confirmation
#4	II.7	Possible ALS	Probable ALS	ALS	EMG shows denervation
#5	II.9	Possible (suspected) ALS	Possible ALS	Likely ALS	Limited spread; no EMG; incomplete workup
#6	–	Definite ALS	Definite ALS	ALS	Bulbar onset; rapid generalization
#7	–	Not ALS	Not ALS	No ALS	Pure LMN disease (segmental PMA)
#8	–	Probable/Definite ALS	Definite ALS	ALS	Typical ALS with ventilatory failure
#9	–	Not classifiable	Not classifiable	Not classifiable	No data available

We assessed the consequences of p.N598S at the protein level using skin biopsy-derived fibroblast cultures and autopsy tissue (cerebellum) from patients #2 and #6 with the p.N598S mutation. Comparing the NEK1 protein levels in these samples using ELISA and Western Blot, and comparing them to tissue from ALS patients carrying the known ALS-associated truncating NEK1 LoF variants p.R812* and p.S1036* (^4^), sporadic ALS probands, or matched controls, we found that the p.N598S variant does not reduce NEK1 protein expression (Fig. 1D–E). Further neuropathological examination revealed that patient #6 with p.N598S, just like a patient with the truncating p.R812* variant, exhibited classical cytoplasmic neuronal pTDP-43 inclusions in the anterior horn of the spinal cord (Fig. 1F) and, to a lesser extent, in the motor cortex (Fig. 1G) and brainstem (Fig. 1H).

Collectively, p.N598S exhibited a Mendelian inheritance pattern, was enriched in ALS cohorts, and displayed classical ALS neuropathology.

**Fig. 2:**
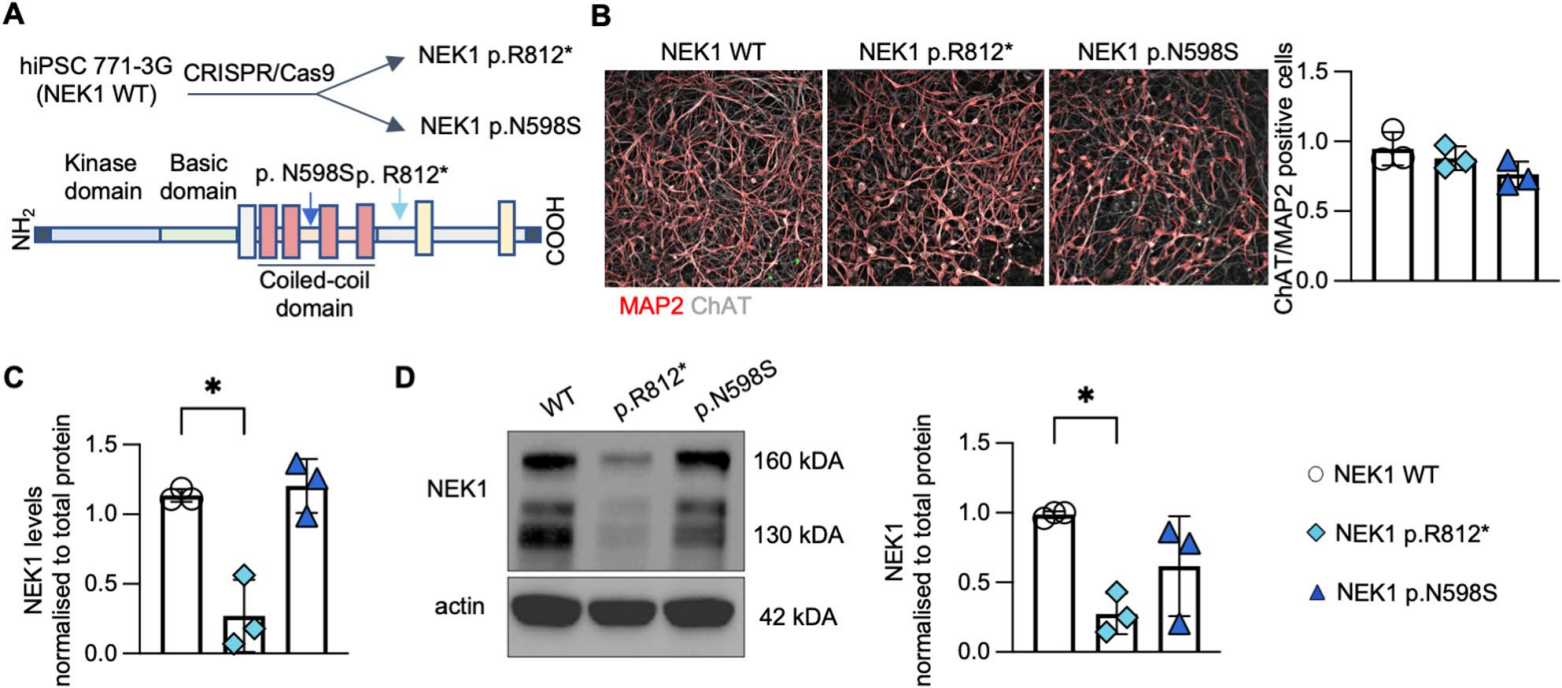
p.N598S shows normal NEK1 protein expression in isogenic motor neuron cultures.** A** Scheme of generation of CRISPR/Cas9-generated isogenic iPSC lines carrying the NEK1 p.R812* and p.N598S variants including their localization in the protein. **B** Fluorescence images of DIV42 iPSC-derived motor neurons show no differences in MAP2 (red) and ChAT (grey) protein expression levels compared to controls. **C**,** D** Quantification of NEK1 protein levels in DIV21 iPSC-derived motor neurons from CRISPR/Cas9-edited lines using ELISA and Western Blot. Mean ± SD of *n* = 3 independent differentiations; statistical significance was determined by one-way ANOVA followed by pairwise comparisons with* p*-values adjusted using the Benjamini–Hochberg false discovery rate (FDR) method; *p* < 0.05 (*)

Since the p.N598S variant maintained normal NEK1 protein expression in patients´ biopsies, we hypothesised that this disease-causing variant could be helpful to clarify the downstream pathomechanisms of NEK1-ALS. To isolate the effect of the p.N598S variant in a genetically controlled context and minimise the impact linked to inter-patient variability, we used an isogenic background and compared it to the ALS-associated pathogenic truncating variant p.R812* [4, 12]. Using CRISPR-Cas9, we generated hiPSC lines carrying either the p.N598S or the p.R812* variant from a line obtained from a healthy donor (NEK1 WT) (Fig. 2A). Using a standardised protocol [13], all three lines (p.N598S, p.R812*, and WT) differentiated efficiently into spinal motor neurons (MNs), and the resulting cultures displayed comparable cellular compositions at day in vitro (DIV) 21 (Fig. 2B). This indicates that neither NEK1 variant impairs MN differentiation. Consistent with patient data (Fig. 1D–E), protein quantification by ELISA (Fig. 2C) and Western blot (Fig. 2D) confirmed that p.N598S-mutant MNs maintain NEK1 protein levels comparable to WT.

**Fig. 3:**
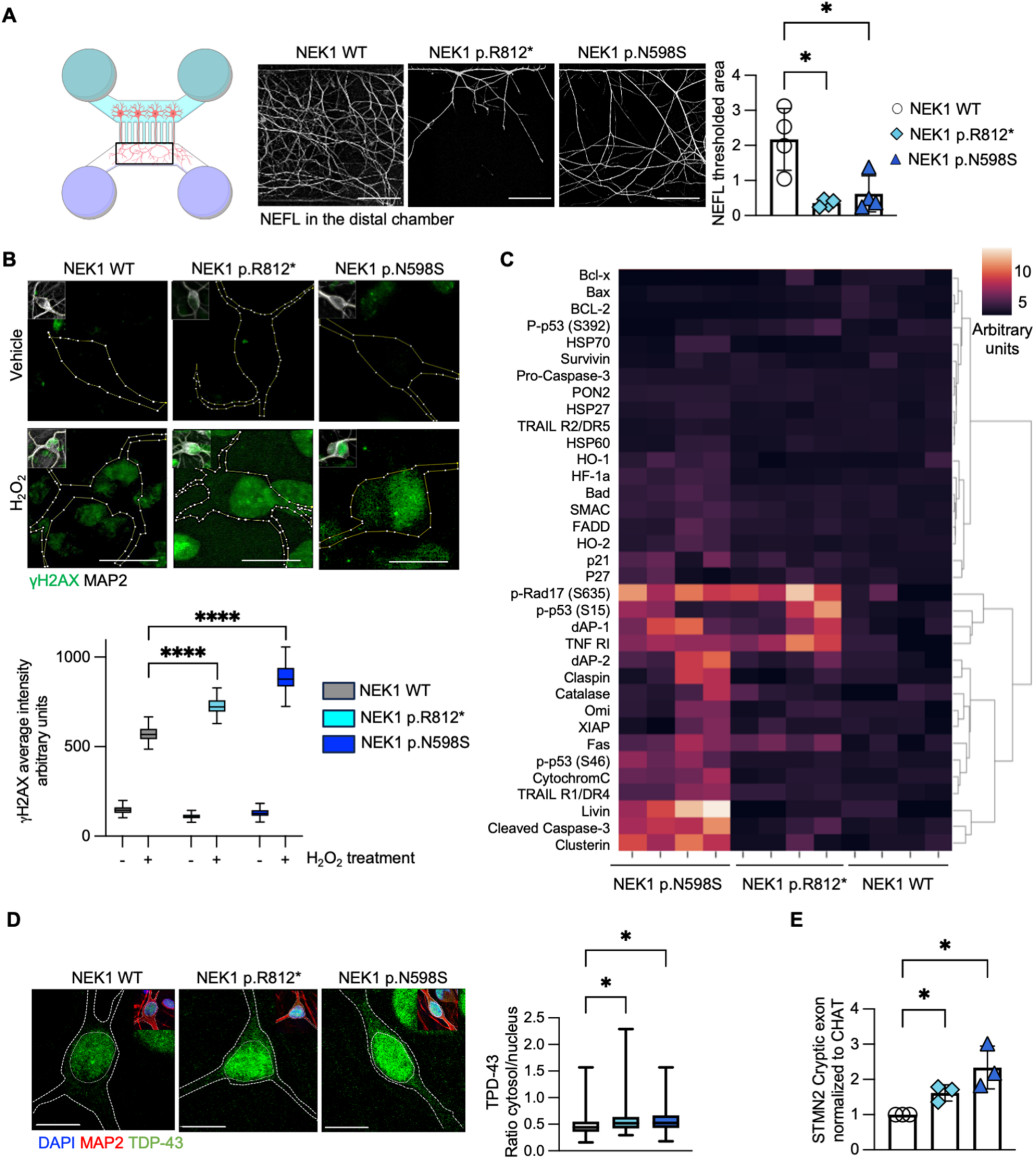
The p.N598S variant causes pathological cellular phenotypes associated with NEK1 haploinsufficiency in cultured human motor neurons.** A** Scheme of the microfluidic chamber setup: cells were plated on the proximal side (blue), and axonal outgrowth was assessed on the distal side (white) and fixed at DIV28. Representative images from the distal compartment (highlighted in black in the scheme) are shown, along with quantification of the NfL-positive area (white) using threshold analysis. Scale bar: 500 μm. Mean ± SD of *n* = 4 independent differentiations; statistical significance was determined by one-way ANOVA followed by pairwise comparisons with p-values adjusted using the Benjamini–Hochberg false discovery rate (FDR) method; *p* < 0.05 (*). **B** Fluorescence images and quantification of motor neurons at DIV42 stained against γH2AX (green), before and after chemically induced oxidative stress using H_2_O_2_. Mean ± SD of *n* = 3 independent differentiations; statistical significance was determined by two-way ANOVA with post hoc pairwise comparisons adjusted for multiple testing (FDR); *p* < 0.0001 (****). **C** Hierarchical clustered heatmap of apoptotic protein array expression profiles in NEK1-mutant motor neurons at DIV21. **D** Representative images and analysis of TDP-43 nucleocytoplasmic translocation (green) from the nucleus to the cytoplasm at DIV42. Scale bar: 10 μm. Mean ± SD of *n* = 3 independent differentiations; statistical significance was determined by one-way ANOVA followed by pairwise comparisons with p-values adjusted using the Benjamini–Hochberg false discovery rate (FDR) method; *p* < 0.05 (*). **E** qPCR-based quantification of *STMN2* cryptic exon inclusion at DIV42. Mean ± SD of *n* = 3 independent differentiations; statistical significance was determined by one-way ANOVA followed by pairwise comparisons with p-values adjusted using the Benjamini–Hochberg false discovery rate (FDR) method; *p* < 0.05 (*)

We next investigated whether the p.N598S variant is sufficient to trigger established pathological phenotypes associated with NEK1 haploinsufficiency in human MNs [8–10]. To this end, we cultured MNs from three isogenic lines in microfluidic chambers and examined axonal morphology at DIV42 using immunostaining for the axonal protein Neurofilament light chain (NEFL/NfL). Quantification of the NfL-positive area in the distal chamber compartment revealed a reduction in both p.N598S- and p.R812*-mutant MNs compared to WT, indicating an axonopathy in both variants (Fig. 3A). Following H_2_O_2_ treatment, both p.N598S- and p.R812*-mutant MNs exhibited increased DNA damage, reflected by higher γH2A.X+ foci intensity relative to WT (Fig. 3B). In addition, a targeted protein array confirmed elevated levels of apoptotic proteins in NEK1-mutant cultures compared to controls (Fig. 3C).

The nucleocytoplasmic translocation of TDP-43 leads to a loss of its nuclear function, resulting in the mis-splicing of cryptic exons in TDP-43 target transcripts, including STMN2. TDP-43 pathology represents a central hallmark of both sporadic and familial ALS, including NEK1-associated ALS (Fig. 1F–H) [11]. To investigate whether this mechanism is present in our model, we analysed TDP-43 in the three previously described isogenic motor neuron (MN) cultures at DIV42. Although TARDBP expression levels were comparable across all lines (Supplementary Fig. 2), NEK1-mutant cultures exhibited an increased cytoplasmic-to-nuclear TDP-43 ratio (Fig. 3D), indicating aberrant TDP-43 localization. Consistent with this observation, these cultures also showed elevated levels of cryptic STMN2 transcripts (Fig. 3E), a feature not previously reported in NEK1-ALS. To further assess the impact of this novel NEK1 missense variant on TDP-43 pathology, we induced TDP-43 dysfunction in HEK cells overexpressing either NEK1-WT or the p.N598S variant using the proteasome inhibitor bortezomib. While bortezomib treatment led to an accumulation of cryptic *STMN2* transcripts in both conditions, this increase reached statistical significance only in cells expressing the p.N598S variant (Supplementary Fig. 3). These findings further support a detrimental role of this variant in exacerbating TDP-43-associated pathology.

**Fig. 4:**
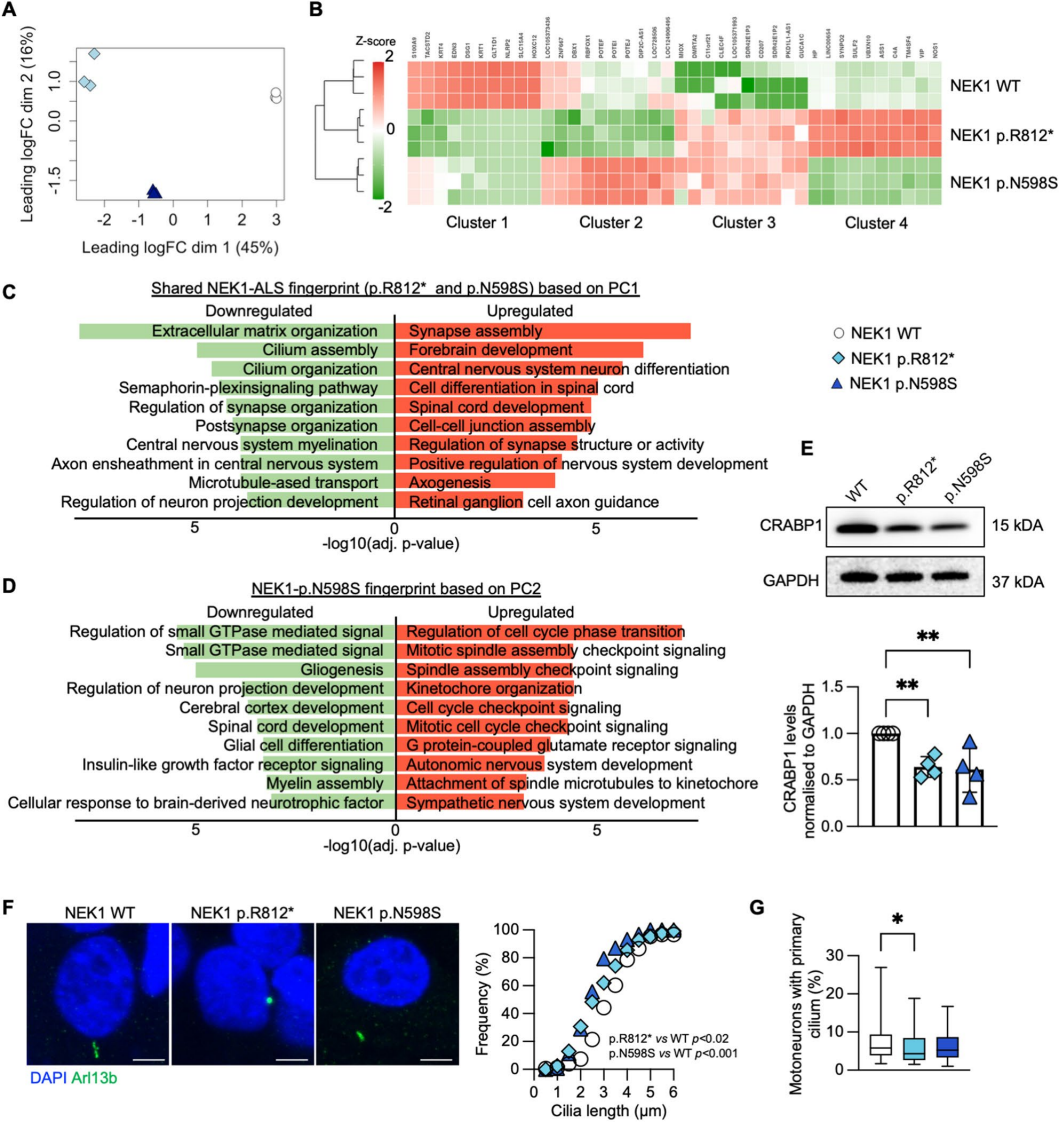
The p.N598S variant shares transcriptomic signatures and ciliary alterations associated with NEK1 haploinsufficiency.** A** Multidimensional scaling analysis reveals separation between the three motor neuron lines with different genotypes. **B** Representative hierarchical cluster map based on the top 10 dysregulated genes between genotypes. Colour intensity represents the Z-score, with red indicating positive values (upregulation) and green indicating negative values (downregulation). Cluster 1: shared downregulated genes for NEK1 p.R812* and p.N598S; Cluster 2: specific upregulated transcripts for NEK1 p.N598S; Cluster 3: shared upregulated genes; Cluster 4: genes specifically downregulated in NEK1 p.N598S. **C** Gene Ontology (Biological process) enrichment analysis of genes commonly upregulated (red) and downregulated (green) in both an NEK1 p.R812* and p.N598S. **D** Gene Ontology (Biological process) enrichment analysis of genes specifically upregulated (red) and downregulated (green) in NEK1 p.N598S-mutant compared to p.R812* motor neurons. **E** Western blot analysis showed decreased expression of CRABP1 protein in NEK1-mutant samples at DIV38. Mean ± SD of *n* = 4 independent differentiations; statistical significance was determined by one-way ANOVA followed by pairwise comparisons with p-values adjusted using the Benjamini–Hochberg false discovery rate (FDR) method; *p* < 0.01 (**). **F** Representative images and analysis of cilia length at DIV38. Cells were stained with DAPI (blue) and Arl13b (green) to assess ciliary morphology; quantification confirmed a significant reduction in cilia length in both mutants. Scale bar: 10 μm. Mean of *n* = 4 independent differentiations; statistical significance was determined by Kruskal–Wallis test followed by Dunn’s post hoc test with FDR correction for multiple comparisons. **G** Quantification of the percentage of motor neurons with primary cilia in NEK1-WT, p.R812* and p.N598S cultures. *N* = 4 independent differentiations; statistical significance was determined by Kruskal–Wallis test followed by Dunn’s post hoc test with FDR correction for multiple comparisons.

We next performed RNA sequencing on cultured human motor neurons to determine how the p.N598S variant alters the transcriptome. Multidimensional scaling (MDS) analysis (PCA) revealed that dimension 1 (dim1) clearly separated both NEK1-mutant lines from wild-type (WT) samples (Fig. 4A), indicating the presence of a shared NEK1-dependent transcriptional program. In contrast, dim2 distinguished p.N598S from p.R812* cultures, highlighting variant-specific transcriptional signatures. Hierarchical clustering based on the top 500 down- and upregulated genes (ranked according to their correlation coefficient between the two dimensions; Supplementary Table 2) further supported these findings, with clusters 1 and 3 representing commonly down- and upregulated transcripts across both mutants, and clusters 2 and 4 capturing variant-specific differences (Fig. 4B). We found that the genes associated with dim1 and defining a “NEK1-ALS fingerprint” were associated with pathways related to cilium function, synaptic processes, axonal biology, and central nervous system development and differentiation (Fig. 4C). Variant-specific changes associated with dim2 showed that p.N598S cultures exhibited downregulation of genes involved in GTPase signalling, spinal cord and cortical development, insulin-like growth factor receptor signalling, and myelination. In contrast, genes upregulated in p.N598S were predominantly linked to cell cycle checkpoint control and kinetochore dynamics (Fig. 4D).

Together, these results define a shared NEK1-ALS transcriptional signature while also revealing distinct, variant-specific effects in human motor neurons. Notably, genes related to primary cilium function were prominently represented within the common transcriptional fingerprint. In line with this, both p.N598S and p.R812* cultures showed reduced levels of CRABP1 (Fig. 4E), a protein previously associated with ciliary dysfunction [14]. Furthermore, neurons carrying either variant exhibited significantly shorter primary cilia compared to WT controls (Fig. 4F). However, only the loss-of-function variant (p.R812*) led to a significant reduction in the proportion of ciliated neurons (Fig. 4G), suggesting that the two NEK1 variants differentially affect ciliogenesis versus ciliary maintenance or maturation. Importantly, we did not detect any significant differences in the interaction between C21orf2 and either NEK1-WT or the p.N598S variant (Supplementary Fig. 4). This indicates that the observed phenotypes are unlikely to result from disrupted protein–protein interaction or reduced NEK1 levels. Instead, our data support the notion that impaired kinase activity might be key driver of ciliary defects and, more broadly, of the diverse cellular phenotypes associated with this novel NEK1 missense variant.

**Fig. 5:**
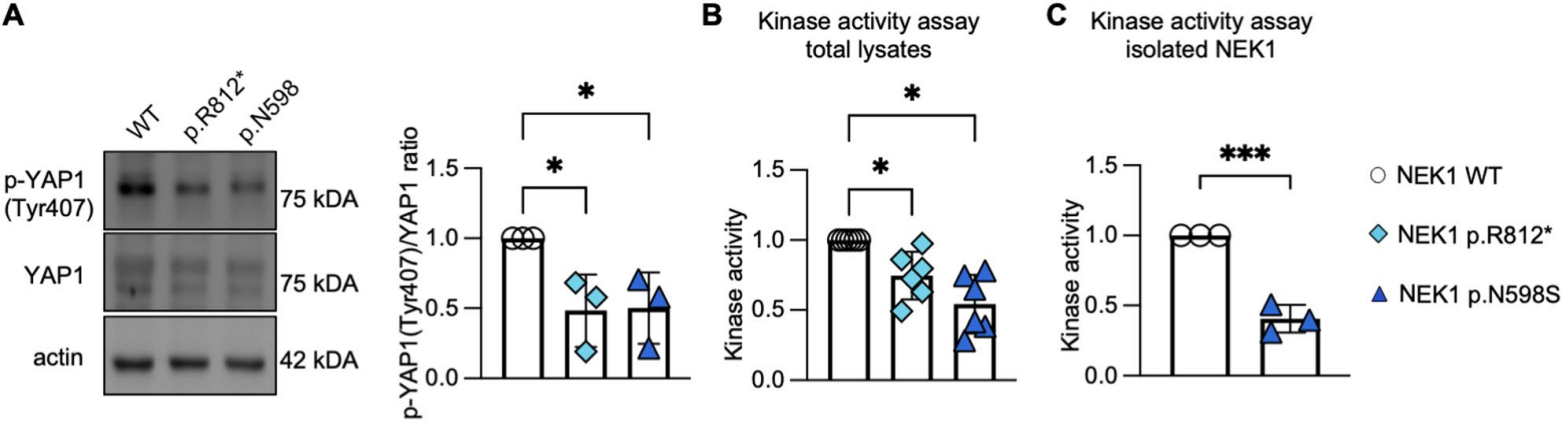
The p.N598S variant impairs NEK1 kinase function.** A** Representative Western Blot and quantification of phosphorylated YAP1 (p-YAP1, Tyr407) at DIV42, showing reduced phosphorylation in NEK1 mutants. Mean ± SD of *n* = 3 independent differentiations; statistical significance was determined by one-way ANOVA followed by pairwise comparisons with* p*-values adjusted using the Benjamini–Hochberg false discovery rate (FDR) method; *p* < 0.05 (*). **B** Kinase activity assays from total lysates at DIV42 show reduced kinase activity in both NEK1 p.R812* and p.N598S. Mean ± SD of *n* = 6 independent differentiations; statistical significance was determined by one-way ANOVA followed by pairwise comparisons with* p*-values adjusted using the Benjamini–Hochberg false discovery rate (FDR) method; *p* < 0.05 (*). **C** Kinase activity assay of extracted NEK1 WT and NEK1 p.N598S at DIV42 shows reduced kinase activity in mutant. Mean ± SD of *n* = 3 independent differentiations; statistical significance was determined by one-way ANOVA followed by pairwise comparisons with* p*-values adjusted using the Benjamini–Hochberg false discovery rate (FDR) method; *p* < 0.001 (***)

Notably, while NEK1 kinase activity has been proposed as the principal pathomechanism in NEK1-ALS [8], this hypothesis still required in vivo confirmation. NEK1 p.N598S MN cultures exhibited increased susceptibility to DNA damage and ciliary abnormalities (Fig. 3B, Fig. 4C-G), both processes known to depend on NEK1 kinase function [9, 15]. This led us to hypothesize that the p.N598S variant might directly impact NEK1 kinase activity. To test this, we quantified the phosphorylation of the established direct NEK1 target YAP1 at tyrosine 407 (pTyr407) [16]. Both p.N598S- and p.R812*-mutant MN cultures showed reduced YAP1 pTyr407 levels compared with their isogenic counterpart (Fig. 5A), supporting reduced kinase activity. This was further assessed in whole lysates from MN cultures, and in immunopurified NEK1-WT and p.N598S. Overall kinase activity was markedly reduced in both p.N598S- and p.R812*-mutant cultures compared to wild-type MNs (Fig. 5B). Direct measurement of purified NEK1 confirmed that the p.N598S variant displayed reduced kinase activity compared with wild-type NEK1 (Fig. 5C).

**Fig. 6:**
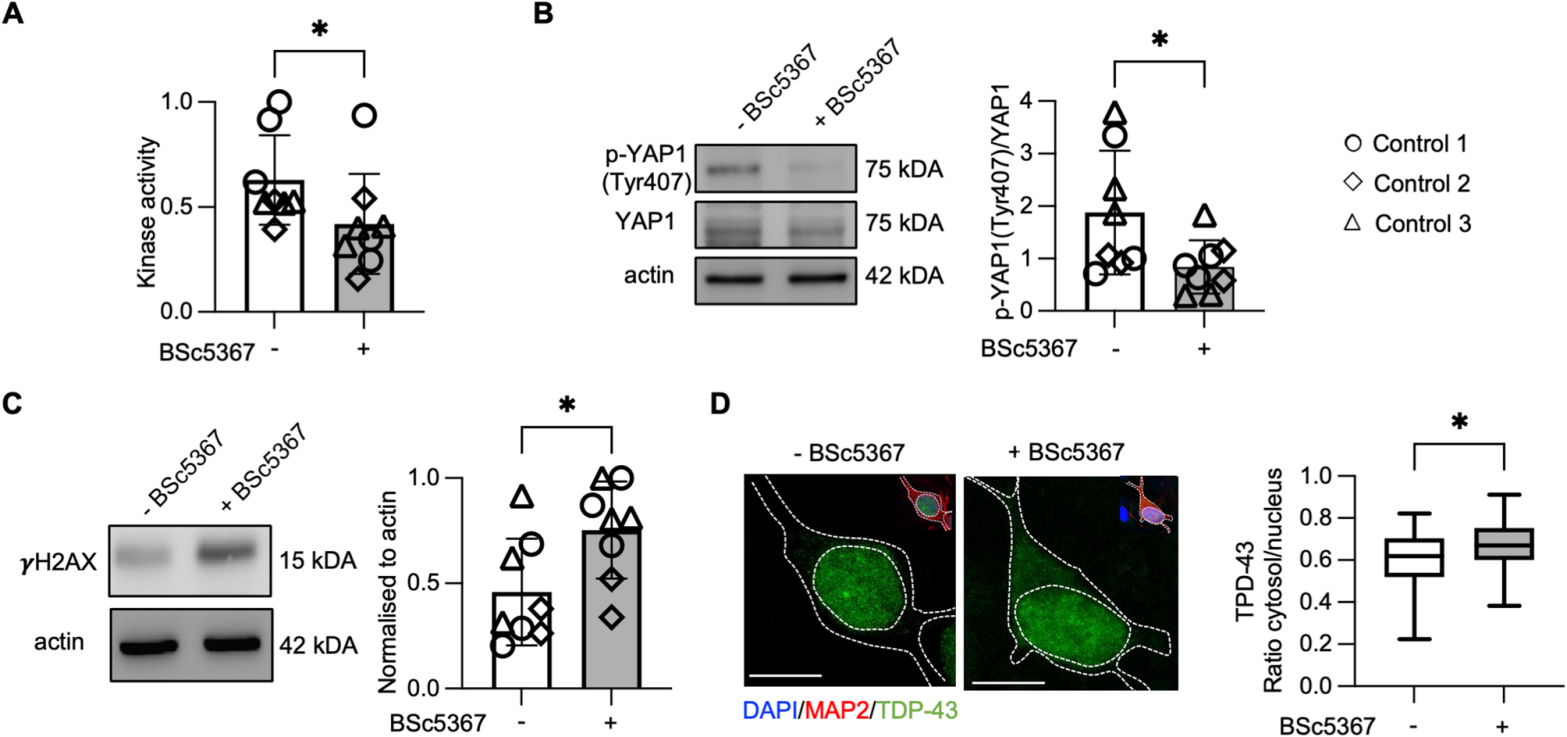
NEK1 inhibition recapitulates established pathological phenotypes associated with NEK1-ALS in cultured motor neurons.** A** Kinase activity assay of total lysates at DIV42 confirms reduced kinase activity after NEK1 inhibition. Mean ± SD of *n* = 3 independent differentiations; statistical significance was determined by one-way ANOVA followed by pairwise comparisons with* p*-values adjusted using the Benjamini–Hochberg false discovery rate (FDR) method; *p* < 0.05 (*). **B** Representative Western blot and quantification of p-YAP1 (Tyr407) at DIV42 show reduced phosphorylation after treatment with the NEK1 inhibitor. Mean ± SD of *n* = 3 independent differentiations; statistical significance was determined by one-way ANOVA followed by pairwise comparisons with p-values adjusted using the Benjamini–Hochberg false discovery rate (FDR) method; *p* < 0.05 (*). **C** Representative Western blot and quantification of γH2AX at DIV42 indicate increased DNA damage following inhibitor treatment. Mean ± SD of *n* = 3 independent differentiations; statistical significance was determined by one-way ANOVA followed by pairwise comparisons with p-values adjusted using the Benjamini–Hochberg false discovery rate (FDR) method; *p* < 0.05 (*). **D** Increased nucleocytoplasmic translocation of TDP-43 at DIV42 observed following NEK1 inhibitor treatment, consistent with ALS pathology. Scale bar: 10 μm. Mean ± SD of *n* = 3 independent differentiations; statistical significance was determined by one-way ANOVA followed by pairwise comparisons with p-values adjusted using the Benjamini–Hochberg false discovery rate (FDR) method; *p* < 0.05 (*)

To further establish causality, we treated MNs derived from three healthy donors with the NEK1-specific inhibitor BSc5367. Exposure to 11.5 nM BSc5367 for 48 h significantly reduced kinase activity (Fig. 6A), decreased YAP1 phosphorylation at Tyr407 (Fig. 6B), and increased γH2A.X levels (Fig. 6C). Most notably, pharmacological inhibition of NEK1 was also sufficient to induce TDP-43 nucleocytoplasmic translocation in control MNs (Fig. 6D).

Altogether, our analyses provide strong evidence for pathogenicity of the NEK1 p.N598S variant, supported by extreme rarity (PM2), significant enrichment in ALS cases (PS4), robust functional deficits in motor neurons (PS3), co-segregation with disease (PP1), phenotype consistency (PP4), and limited but supportive computational predictions (PP3) (Table 3). Consequently, according to the ACMG/AMP guidelines with consideration of ClinGen recommendations for functional evidence and case–control data the p.N598S variant is classified as likely pathogenic (PS3 + PS4 + PM2 + ≥ 2 supporting criteria).

**Table 3:** ACMG/AMP variant classification of the NEK1 variant c.1793 A > G; (p.Asn598Ser)

Domain	Criterion	Strength	Evidence from this study
Functional studies	PS3	Strong	Multiple orthogonal assays in isogenic human motor neurons show reduced NEK1 kinase activity, decreased YAP1 phosphorylation, increased DNA damage, apoptosis, ciliary defects, and TDP-43 nucleocytoplasmic translocation; pharmacological NEK1 inhibition phenocopies these effects
Case enrichment	PS4	Strong	Significant enrichment in ALS cases vs. controls (OR = 10.7; 95% CI 4.15–27.42; *p* = 0.00019)
Population data	PM2	Moderate	Low allele frequency in gnomAD (MAF 2.65 × 10^–5^ in European population)
Segregation data	PP1	Supporting to Moderate	Co-segregation with disease in multiple affected family members (Fig. 1A)
Computational evidence	PP3	Supporting	SIFT: deleterious (low confidence); PolyPhen: possibly damaging; CADD: 24.8
Clinical data (Phenotype specificity)	PP4	Supporting	Majority of p.N598S-mutant patients exhibit spinal-onset, slowly-progressive ALS consistent with NEK1-ALS [20–24]
Overall classification	Likely Pathogenic (PS3 + PS4 + PM2 + ≥ 2 supporting criteria)		

In conclusion, our findings provide genetic and functional evidence that disruption of NEK1 kinase function constitutes a central and sufficient disease mechanism in NEK1-associated ALS. We show that NEK1 kinase dysfunction drives established phenotypes of NEK1 haploinsufficiency in cultured human motor neurons. These findings not only provide a valuable new model for NEK1-ALS but also establish a framework for functional testing of variants of uncertain significance, highlighting restoration of kinase activity as a key pharmacological target for therapeutic intervention.

## Discussion

Here, we demonstrate that the kinase-dead NEK1 missense variant p.N598S meets ACMG/AMP criteria for likely pathogenicity and provides convergent genetic and functional evidence supporting a disease-causing role in NEK1-associated ALS.

To date, only truncating loss-of-function (LoF) variants in *NEK1* have been shown to be causative of ALS. Our research presents the first instance of a rare disease-causing NEK1 missense variant, p.N598S, which co-segregates with ALS. While the single-generation pedigree limits our definitive interpretation, the variant’s low frequency, high evolutionary conservation, and enrichment within the ALS population, as well as the observed disruption of kinase function support its classification as likely pathogenic under ACMG/AMP criteria. For truncating variants in *NEK1*, as it is the case for most familial ALS-causing genes, incomplete penetrance is the rule rather than the exception [4, 5, 17]. Of note, the p.N598S variant seems to exhibit a higher penetrance than any previously reported *NEK1* variant including truncating LoF variants. We propose two potential explanations for this. First, a dominant-negative effect of p.N598S may increase its pathogenicity and penetrance. This is supported by our findings in induced motor neuron cultures, which show more severe phenotypes—including exacerbated DNA damage accumulation and apoptosis—compared to the truncating pR812* variant. Second, environmental co-factors, such as exposure to DNA damage, may contribute to the variant’s penetrance. The high prevalence of smoking in the affected family, a known risk factor for ALS [18], aligns with this hypothesis. Additionally, the presence of multiple cancers in two p.N598S-positive family members is consistent with preclinical evidence that NEK1 haploinsufficiency may predispose individuals to neoplasia [19]. Ultimately, it remains unclear whether the observed increase in penetrance of p.N598S—compared to truncating NEK1 variants—is intrinsic to the variant itself.

Most patients with suspected or manifest ALS carrying the p.N598S variant described in our study exhibited a rather slow disease progression and survival clearly above average in ALS. The finding of spinal lower motor-neuron predominance and often slower disease progression is in line with previous case reports on patients with pathogenic *NEK1* LoF variants observing an overly high prevalence of spinal onset and flail-arm syndrome [20–24].

The NEK1 p.N598S missense variant was sufficient to induce known established NEK1-ALS-associated phenotypes in vitro and in vivo including impaired axonal outgrowth, enhanced susceptibility to DNA damage, increased apoptosis, and ciliary dysmorphia as well as TDP-43 pathology [8, 10–12]. We found that cytoplasmatic translocation of TDP-43 is linked to impaired kinase activity of NEK1. Several mechanisms may explain this association: (i) defective nuclear import [8], (ii) impaired autophagy [25], and (iii) altered stress granule formation [26]. Concerning the latter process, recent evidence demonstrates that YAP1, a direct phosphorylation target of NEK1 (at pYAP-Tyr407) [16], directly interacts with TDP-43, promoting its homotypic multimerization and phase separation, while at the same time preventing hyperphosphorylation and pathological solidification under stress conditions [26]. The link between NEK1 kinase activity and TDP-43 nucleocytoplasmic translocation suggests NEK1 signalling as a potential therapeutic target also for other ALS forms linked to TDP-43 pathology.

In motor neuron models relevant to ALS, ALS-associated variants in *NEK1* (truncating) or *C21ORF2* have been associated with altered ciliary structure, reduced ciliation frequency and impaired cilia-dependent Sonic Hedgehog signalling [10, 14]. These defects were accompanied by altered tubulin acetylation, calcium dysregulation, and activation of Aurora kinase A–HDAC6 pathways, promoting ciliary disassembly [10]. Overall, this evidence supports the idea that ciliary defects are a reproducible cellular consequence of NEK1 perturbation and may contribute to ALS pathogenesis, potentially by disrupting signaling pathways critical for motor neuron survival. However, a direct causal link between ciliary dysfunction and neurodegeneration in NEK1-associated ALS has not yet been fully established. Importantly, not all NEK1 variants have been functionally characterized, and the extent to which kinase activity versus other domains of the protein contribute to ciliary phenotypes had remained unclear. Thus, the key novelty of our study in this context is that we demonstrate a rare, ALS-associated missense NEK1 variant (N598S) with preserved protein expression and reduced kinase activity recapitulates these previously established ciliary phenotypes which strongly indicates that they are kinase-dependent.

Our work demonstrates both genetically (through the kinase-dead p.N598S variant) and pharmacologically (by using the NEK1 inhibitor BSc5367) that NEK1 kinase dysfunction acts upstream of the pathological phenotypes of NEK1-associated ALS in cultured human motor neurons. The p.N598S variant is, to our knowledge, the only NEK1 missense variant linked to ALS that has been shown to markedly impair kinase activity. Notably, two cases of short-rib thoracic dysplasia 6 with or without polydactyly, a ciliopathy, have been submitted to Clinvar (Clinvar variation ID: 348110), which further corroborates the pathogenicity and disruption of kinase function of the p.N598S variant since ciliary morphology depends on NEK1 kinase activity [9]. It is conceivable that there are missense variants in *NEK1* that do not affect protein expression or kinase function but nevertheless cause ALS by disruption of a certain function or interaction critical for disease causation. Missense variants causing such an isolated functional defect have indeed already been reported in NEK1-associated ciliopathies. The NEK1 p.D1277A variant causes axial spondylometaphyseal dysplasia in a compound-heterozygous fashion together with the ALS-associated LoF variant p.S1036* [27]. p.D1277A abolishes the interaction of NEK1 with C21ORF2 (encoded by another ALS gene) which results in defective ciliogenesis [9]. c.3584-10T>A has been reported to cause lethal short-rib polydactyly syndrome in homozygous condition [28]. This variant leads to a NEK1 protein lacking a C-terminal fragment which abolishes the interaction with the VDAC1 channel, also critical for ciliogenesis [28]. Our study, and these examples, underline that the molecular in-depth characterization of disease-associated rare missense variants by means of a combinatorial analysis of disease-relevant cellular models and patient tissue is of great importance for the education of the disease-determining pathomechanisms and thus informs diagnostic and therapeutic targets and strategies.

In summary, we provide genetic and functional evidence that heterozygous disruption of NEK1 kinase function is sufficient to cause ALS. Therefore, kinase dysfunction appears to be the disease-defining pathomechanism underlying NEK1-ALS acting upstream of the known pathological phenotypes observed in NEK1-mutant motor neurons, including cytoskeletal perturbation, ciliary dysmorphia, DNA damage, and apoptosis. Our results pave the way for the classification of genetic variants of uncertain significance in *NEK1* through assessment of NEK1 kinase activity. Not least, they highlight the restoration of kinase activity as the prime pharmacological target for therapeutic rescue of NEK1-associated ALS.

## Methods

### Ethics approval and consent to participate

All clinical data and biosamples were obtained from patients with written informed consent. The studies were approved by the institutional review boards of the University of Ulm and the University Medical Center Utrecht. Collection of patients’ material including blood, skin biopsy and autopsy samples was performed in the context of the registry and trace study “German Network for Motor Neuron Diseases (MND-NET)” (approval nr. 19/12 from the Ethics Committee of Ulm University). The use of iPSC models for ALS studies was approved by the Ethics Committee of KU Leuven (approval nr. S67294). The use of autopsy tissue for ALS studies was approved by the ethics committee of the medical faculty of the RWTH Aachen University (approval nr. 127/18).

### Patient information

#### Family of Fig. 1A

Patient II.2 experienced disease onset at 62 years of age, accompanied by pronounced weight loss. Motor symptoms were present alongside cognitive impairment, with a last ECAS score of 58. The disease progressed rapidly to tetraparesis, bulbar palsy, and ventilatory insufficiency. He died 1.5 years after symptom onset.

Patient II.5 (the index patient) had hand-onset ALS at age 55. Her disease progression has been slow, and she remains alive five years after onset. Currently, she exhibits combined amyotrophic and spastic tetraparesis, mild pseudobulbar palsy, and ventilatory insufficiency, requiring non-invasive mechanical ventilation.

Patient II.6 initially presented with a gait disorder due to spastic palsy of the lower limbs. He shows slow disease progression and remains alive 6.5 years after onset. Over time, he developed pseudobulbar palsy and cognitive impairment, with a last ECAS score of 41.

Patient II.7 first noticed leg paresis at age 55, particularly after prolonged exercise, along with occasional fasciculations in both calves. Neurological examination revealed mild paralysis of both upper extremities and hyperreflexia in both arms and the left leg. Electromyography demonstrated acute and chronic denervation in two of four muscles examined, suggesting early, slowly progressive ALS.

Family member II.9 exhibits widespread fasciculations, mild weakness of left arm flexion, an elevated left radial reflex, and minimal atrophy of both hands, raising suspicion of early ALS. He declined further diagnostic evaluation.

Notably, patients II.5 and II.6 have comorbid autoimmune diseases (II.5: membranous glomerulonephritis; II.6: suspected primary progressive multiple sclerosis), and all affected family members were or are heavy smokers. Additionally, II.5 and II.9 have comorbid chronic obstructive pulmonary disease (COPD).

The parents of affected family members showed no clear signs of motor neuron disease. The p.N598S variant was most likely transmitted by the father (I.1), as this variant is also present in another branch of his lineage, in which no cases of motor neuron disease have been reported (data not shown). I.1 died at age 90 from multiple cancers (stomach and skin), while his wife (I.2) died at age 79. Of note, family member II.4, who is unaffected by ALS, also has a history of multiple cancers (bladder, skin, and lung).

#### Other cases

Case #6 (Table 1) presented with bulbar onset at 80 years of age and exhibited a classical phenotype, characterized by rapid disease progression to tetraplegia and cachexia within six months. She was a non-smoker, and her family history was negative for motor neuron disease (MND). She died 11 months after disease onset.

Patient #7 was diagnosed with “segmental spinal muscular atrophy,” presenting with slightly asymmetric amyotrophic weakness and fasciculations in the upper limbs. She remains alive more than 12 years after symptom onset.

Patient #8 experienced typical ALS, progressing to require tube feeding and non-invasive ventilation within 2–3 years of onset. Detailed clinical information for case #9 is not available.

Also see Table 2 for alignment with various diagnostic ALS criteria.

### Genetic analysis

*C9orf72* genotyping in all samples was carried out by fragment analysis and repeat-primed PCR [2]. All normal homozygotes and expanded alleles were confirmed with Southern blot. Panel analysis based on targeted gene sequencing (custom Illumina panel) was performed in cases #1, #3, and #6 (Table 1) as previously described [2]. Panel analysis was based on WES in case #2 and on WGS in cases #7–9. Genotyping by PCR for c.1793 A > G (p.Asn598Ser) was performed for cases #4 and #5.^1^ The ALS-associated gene panel included the following ALS/FTD genes: *ALS2*,* ANG*,* ARHGEF28*,* ATXN2*,* BSCL2*,* C9orf72*,* CCNF*,* CHCHD10*,* CHMP2B*,* DCTN1*,* ERBB4*,* FIG4*,* FUS*,* GBE1*,* GLE1*,* GRN*,* HNRNPA1*,* HNRNPA2B1*,* HSPB1*,* HSPB8*,* KIF5A*,* MAPT*,* MATR3*,* MME*,* NEFH*,* NEK1*,* OPTN*,* PFN1*,* PRPH*,* SETX*,* SIGMAR1*,* SOD1*,* SPG11*,* SPG20*,* SQSTM1*,* TAF15*,* TARDBP*,* TBK1*,*TUBA4A*,* UBQLN2*,* VAPB*,* VCP*,* VEGFA*,* VPS54.*

### Pedigree

The online tool “QuickPed” was used to draw the pedigree in Fig. 1A [29].

### Kinship analysis

Kinship analysis was performed using KING on HapMap Phase 3 SNPs in PLINK2 (www.cog-genomics.org/plink/2.0/) format. The relatedness cutoff was set to a kinship coefficient < 0.0442.

### Variant classification

Variant classification of the variant NM_001199397.3(NEK1):c.1793 A > G (p.Asn598Ser) was performed according to ACMG/AMP guidelines with consideration of ClinGen recommendations for functional evidence and case–control data (see summary in Table 3).

### Fibroblasts culture

Culture of fibroblasts generated from skin biopsies was performed as previously described [12]. Briefly, skin biopsies were plated onto Matrigel^®^-coated 6-well plates (Corning, 354,277), pinned with a sterile needle to the plate bottom and cultured in DMEM/F12 (Thermo Fisher Scientific 10565018) supplemented with 20% knockout serum replacement (Thermo Fisher Scientific10828028), 1% non-essential amino acids (NEAA; Thermo Fisher Scientific 11140050) and 1% antibiotic-antimicotic (Thermo Fisher Scientific 15240062) to let fibroblast outgrowth and proliferation. Fibroblasts were then detached using Trypsin-EDTA (Thermo Fisher Scientific 15400054) and further cultured.

### Generation and culture of isogenic human induced pluripotent stem cells (hiPSCs)

p.N598S- and p.R812*-mutant hiPSCs were generated from a commercially available line (StemRNA™ Human iPSC 771-3G, RCRP005N; Reprocell Inc.) using an isogenic CRISPR/Cas9 approach to introduce the mutations in a controlled genetic background. Guide RNA (CAUACUUUCAGUUGUAGAGA for p.R812* and GAAGUUUGGCUUUAAUCUGU for p.N598S) together with Cas9 were delivered into cells via electroporation. Cells were allowed to recover in culture for 2 days before genome editing efficiency was assessed by Sanger sequencing following PCR amplification using the following primers: for p.R812* TGTGAGAGGGAGGCACTTCT (forward) TCGTGACTTTCAGAGTAAGCGA (reverse) and for p.N598S CAGAATTTCTCTGGAACAAATGGTCT (forward) and TCCATTAACCGGATTTCAGAACT (reverse). Subsequently, single cells were seeded for clonal expansion.

HiPSCs were cultured on Matrigel^®^-coated 6-well plates (Corning, ) at 37 °C (5% CO2, 5% O2) using mTeSR plus medium (Stem Cell Technologies, 100–0274). After reaching 80% of confluence, the colonies were detached using Dispase (Stem Cell Technologies) and passaged in a 1:3 or 1:6 split ratio. Potential mycoplasma contamination was regularly checked using the MycoStrip™ Mycoplasma Detection Kit (Invivogen, rep-mysnc-50; every second week) and MycoAlert^®^ Mycoplasma Detection Kit (Lonza, LT07-318; once a month).

### Differentiation of hiPSCs into spinal motor neurons

We differentiated MNs from hiPSCs using a previously described protocol [13]. Briefly, hiPSC colonies were detached using Dispase (Stem Cell Technologies, 07923) and cultured in suspension in ultra-low attachment flasks T75 for 3 days for the formation of embryoid bodies (EBs) in hESC medium (DMEM/F12 + 20% knockout serum replacement + 1% NEAA + 1% β-mercaptoethanol + 1% antibiotic–antimycotic + SB-431542 10 µM + Dorsomorphin 1 µM + CHIR 99021 3 µM + Purmorphamine 1 µM + Ascorbic Acid 200 ng/µL + 1% B27 + 0.5% N2). On the fourth day, the medium was switched to MN Medium (DMEM/F12 + 24 nM sodium selenite + 16 nM progesterone + 0.08 mg/mL apotransferrin + 0.02 mg/mL insulin + 7.72 µg/mL putrescine + 1% NEAA, 1% antibiotic–antimycotic + 50 mg/mL heparin + 10 µg/mL of the neurotrophic factors BDNF, GDNF, and IGF-1, SB-431542 10 µM, Dorsomorphin 1 µM, CHIR 99021 3 µM, Purmorphamine 1 µM, Ascorbic Acid 200 ng/µL, Retinoic Acid 1 µM, cAMP 1 µM, 1% B27, 0.5% N2). After 5 further days of cultivation EBs were dissociated into single cells with Accutase (Sigma Aldrich) for 10 min and plated onto 24-well µPlates (Ibidi), 12- well or 6-well plates (Corning) pre-coated with Growth Factor Reduced Matrigel (Corning).

### Western blot

Western blot analyses were performed using protein lysates from hiPSC-derived motor neurons, patient fibroblasts, and cerebellar tissue obtained from patient autopsies. Samples were prepared in RIPA buffer supplemented with protease and phosphatase inhibitors, except for NEK1 blots, where cells were lysed in PBS-/- containing 0.5% NP-40. Equal amounts of protein, determined using the Bradford assay, were separated on 10% SDS-PAGE gels and transferred to nitrocellulose membranes using the Trans-Blot Turbo system (Bio-Rad, USA). Membranes were blocked in 5% BSA (prepared in TBS, pH 7.5, with 0.2% Tween-20) for 2 h at room temperature, followed by overnight incubation at 4 °C with primary antibodies (Suppl. Table 1). Following three washes with TBS + 0.2% Tween-20, membranes were incubated with HRP-conjugated secondary antibodies for 2 h at room temperature, then washed again three times. Protein bands were detected using the ECL detection kit (Thermo Fisher Scientific, #32106) and imaged with a ChemiDoc MP Imaging System (Bio-Rad). Band intensities were quantified using GelAnalyzer version 23.1.1, and protein levels were normalized to β-actin as a loading control.

### Immunocytochemistry

Immunostainings were performed as previously described in Aly et al. [30]. Cells were fixed with 4% paraformaldehyde containing 10% sucrose for 10 min, followed by incubation in blocking solution (PBS with 10% goat serum and 0.2% Triton X-100) for 2 h at room temperature. The same solution was used to dilute the primary antibodies, which were applied overnight at 4 °C. A complete list of primary, including dilutions, is provided in (Suppl. Table 1). After that cells were washed three times with PBS, then incubated with secondary antibodies (diluted 1:1000 in PBS) for 2 h at room temperature. Following an additional three PBS washes, cells were mounted using either ProLong™ Gold Antifade Mountant with DAPI (Thermo Fisher Scientific, #P36935) or Ibidi Mounting Medium (Ibidi, #50001).

### Immunohistochemistry

Paraffin Sects.  (3–4 mm thick) were dewaxed, rehydrated, treated with EnVision FLEX Target Retrieval Solution, Low pH (K8005 Dako), and then incubated in EnVision FLEX Peroxidase-Blocking Reagent (SM801 Dako). The sections were then incubated for 30 min with the primary antibodies (rabbit polyclonal Cosmo Bio LTD Anti-phospho TDP43 (TIP-PTD-P03), 1:2000; rabbit polyclonal Sigma Aldrich Anti-p62/SQSTM1 (P0068), 1:1000) in Antibody Diluent (DM830 Dako), followed by DAB visualization (GV82511-2 Dako) according to our validated standard protocols for the use of these antibodies in routine immunohistochemistry of biopsy and autopsy tissue.

### RT-qPCR

Total RNA was extracted from hiPSC-derived motoneurons using the RNeasy Mini Kit (Qiagen, #74104) according to the manufacturer’s instructions, and eluted in 30 µL of RNase-free water (Qiagen, #129112). For general qRT-PCR, one-step reverse transcription and amplification were performed in a single-tube reaction using the QuantiFast™ SYBR Green RT-PCR Kit (Qiagen, #208054) in a total volume of 20 µL, following the manufacturer’s protocol. For alternative splicing analysis, 1 µg of total RNA was reverse-transcribed into cDNA using the QuantiTect Reverse Transcription Kit (Qiagen, #205313). PCR amplification was then carried out using the RT²SYBR Green qPCR Mastermix (Qiagen, #330501) in a 20 µL reaction volume. Thermal cycling conditions for standard qRT-PCR were as follows: 10 min at 55 °C (reverse transcription), 5 min at 95 °C (initial denaturation), followed by 40 cycles of 5 s at 95 °C (denaturation) and 10 s at 60 °C (annealing/extension). Primer sequences used in this study are listed in Suppl. Table 1. Gene expression levels were normalized to GAPDH. All reactions were performed in technical triplicates. Cycle threshold (Ct) values were calculated using Rotor-Gene Q software (version 2.0.2; Qiagen).

### ELISA

For sample preparation, cell pellets were resuspended in 150 µl of lysis buffer (0.5% NP-40 (v/v) solution in DPBS -/-). The lysates were sonicated three times and then centrifuged at maximum speed for 10 min at 4 °C. The supernatant obtained was collected for the assay (NEK1 ELISA kit, #MBS9340866, MyBioSource). The experiment was performed according to the manufacturing protocol. Briefly, blank, standard and sample wells were arranged on a 96-well plate. The wells were incubated with 100 µl of HRP conjugate reagent for one hour at 37 °C. All wells were then thoroughly washed four times with Washing Buffer. Fifty microlitres of Chromogen A and 50 µl of Chromogen B were added to each well and the plate was incubated for 15 min at 37 °C. Finally, 50 µl Stop Solution was added to each well for five minutes. Optical density was then measured at 450 nm using the Cytation-3 Imaging Reader.

### Chemically induced DNA damage, NEK1 inhibitor treatment of iPSC-derived motoneurons

To chemically induce DNA damage, the cells were treated at DIV42 with 2.5 µM H_2_O_2_ for 30 min at 37 °C. BSc5367 (#HY-144425,MCE) was used to inhibit NEK1 kinase activity [31]. The treatment was performed on control cell lines two days prior to DIV42 at a concentration of 11.5nM (corresponding to the IC_50_ value) over two consecutive treatments, for 48 h in total. The cells were then fixed and used for Western Blotting or immunostaining.

### Kinase activity assay

The ADP-Glo Kinase Assay (#V9101, Promega) was used to detect kinase activity according to the manufacturer’s instructions. Briefly, after preparing the standard curve with ADP/ATP variations, the samples were diluted with 4X kinase buffer D to the similar protein concentrations and incubated with 2.5X ATP/substrate mix for 45 min. The ADP detection reaction was then performed with ADP-Glo for 40 min on a shaker, followed by a 30 min detection reagent incubation. Finally, the plates were read using luminescence with an integration time of 0.5 s.

HEK cells were transfected with HA-tagged NEK1-WT and NEK1-N598S plasmids. The following day, cells were washed once with PBS -/- supplemented with protease inhibitor (#11873580001, Roche). Cells were then collected and centrifuged at 1000×g for 8 min at 4 °C. Co-immunoprecipitation was performed using the Pierce™ Co-Immunoprecipitation Kit (#10007D, Invitrogen). The supernatant was discarded, and the cell pellet was gently resuspended and homogenized in 150 µL of lysis buffer containing 1% NP-40, 50 mM Tris-HCl (pH 7.5), 150 mM NaCl, 2 mM EDTA, and protease inhibitors, prepared in RNase-free water. Lysates were incubated on a rotator at 4 °C for 1 h. Protein concentration was determined using the Bradford assay, and samples were adjusted with antibody binding buffer to a total protein concentration of 400 µg. Subsequently, 40 µL of Anti-HA magnetic beads (#88836, Thermo Fisher Scientific) were added to each sample and incubated overnight at 4 °C on a rotator. The following day, samples were washed three times with Wash Buffer using a magnetic rack. Kinases were eluted in RNase-free water by rotating the samples at room temperature for 1 h.

### Apoptosis protein array

The Proteome Profiler Human Apoptosis Array Kit (#ARY009, R&D Systems) was used to perform the protein array. Samples were prepared in 100 µl of RIPA buffer containing Phosphostopp. First, 2 ml of Array Buffer 1 (the blocking agent) was pipetted into each well of the four-well multi-dish, after which the dish was incubated for one hour at room temperature on a shaker. A predetermined amount of cell lysate was added to 1.25 ml of Array Buffer 1 and then added to the plate, after which the plate was incubated overnight at 4 °C on a shaker. The arrays were washed three times for 10 min. The detection antibody cocktail was diluted in 1.5 ml of Array Buffer 2/3 for each array. The arrays were then incubated with the antibody mixture for one hour on a shaker at room temperature, followed by incubation with streptavidin-HRP for a further 30 min on a shaker at room temperature, and finally with Chemi Reagent Mix for one hour. Images of the membranes were captured using the MicroChemi station and analysed using Fiji ImageJ software.

### RNA sequencing

Bulk RNA-seq reads were processed using Trim Galore to remove adapter sequences and low-quality bases, and the cleaned reads were aligned to the human reference genome (GRCh38) with STAR. Gene-level counts were generated from the aligned reads and differential expression analysis was performed in R using limma, with model fitting and empirical Bayes moderation applied to identify genes differentially expressed between conditions. To capture the major axes of transcriptional variation associated with the ALS footprint, multidimensional scaling (MDS) was applied to the normalized gene expression matrix. Pearson correlation coefficients were computed between individual gene expression profiles and the sample coordinates along MDS dimensions 1 and 2. Genes with an absolute correlation ≥ 0.80 were retained and further stratified by direction of association, yielding four gene sets: positively and negatively correlated genes for each dimension. These gene sets were subsequently used as input for over-representation analysis (ORA) using clusterProfiler to identify biological pathways enriched within each transcriptional axis. Functional enrichment analyses were also carried out using fgsea for ranked gene set enrichment analysis to interpret the biological processes associated with the broader expression changes.

### List of antibodies and primers

The complete list of the antibodies and primers used can be found in Suppl. Table 1.

### Statistics

Statistical analyses were performed using GraphPad Prism (version 10.6.0; GraphPad Software). For comparisons involving more than two groups with normally distributed data, one-way or two-way ANOVA was applied, followed by pairwise post hoc tests with p-values adjusted for multiple comparisons using the Benjamini–Hochberg false discovery rate (FDR) method. For comparisons of more than two groups without normal distribution of data, Kruskal–Wallis test followed by Dunn’s post hoc test with FDR correction was used. Data are presented as mean ± SD unless otherwise specified in the figure legends. Statistical significance was defined as *p* < 0.05 (), *p* < 0.01 (), *p* < 0.001 (), and *p* < 0.0001 (**).

## Supplementary Information

Below is the link to the electronic supplementary material.

Supplementary Material 1.
Fig. 1 Conservation of NEK1 p.N598S variant across multiple species.

Supplementary Material 2.
Fig. 2 NEK1 mutations do not alter the expression levels of TARDBP in human motor neurons. qPCR analysis reveals comparable mRNA levels in NEK1-WT p.R812* and p.N598S cultures. N = 3 independent cultures for each genotype.

Supplementary Material 3.
Fig. 3 NEK1 p.N598S exacerbates TDP-43 pathology. Overexpression of the NEK1 p.N598S variant significantly increases the levels of cryptic STMN2 in HEK cells upon treatment with the proteasome inhibitor bortezomib. N = 3 independent cultures for each genotype. Mean ± SD of n = 3 independent differentiations; statistical significance was determined by two-way ANOVA with post hoc pairwise comparisons adjusted for multiple testing (FDR);* p* < 0.05 (*).

Supplementary Material 4.
Fig. 4 The p.N598S variant does not impact the interaction of NEK1 with C21orf2. Co-immunoprecipitation performed in HEK cells overexpressing either NEK1-WT or p.N598S. The levels of C21orf2 interacting with NEK1 are comparable in both groups.Mean ± SD of n = 3 independent experiments.

Supplementary Material 5.
Table 1: List of antibodies and primers used in this study.

Supplementary Material 6.
Table 2: List of the significantly altered genes highlighted by multidimensional scaling.

## Data Availability

The RNAseq source data have been uploaded to the ENA database with the identifier PRJEB98556.

## Abbreviations

ALS: Amyotrophic lateral sclerosis
GnomAD: Genome aggregation database
hiPSC: human induced pluripotent stem cell
LoF: Loss of function
MAF: Minor allele frequency
MN: Motoneuron
MS: Mass spectroscopy
MND: Motor neuron disease
PolyPhen: Polymorphism phenotyping
SIFT: Sorting intolerant from tolerant
SNP: Single-nucleotide polymorphism
WT: Wildtype
