# Correction: A rare missense variant impacting NEK1 kinase function is associated with ALS

**DOI:** 10.1186/s40478-026-02380-1

**Published:** 2026-07-20

**Authors:** David Brenner, Anna Ponomarenko, Iris Petrut, Sofia Beyrle, Matilde Contardo, Isabel Loss, Constantin Radke, Jonas Frank, Eleni Zimmer, Matthias Schlesner, Pascal Achenbach, Wendy Scheveneels, Amr Aly, Hülya Nazlican, Jasper Hesebeck-Brinkmann, Patrick Oeckl, Kathrin Müller, Reiner Siebert, Tobias Böckers, Kristel van Eijk, Jan Veldink, Alexander Kleger, Medhanie Mulaw, Peter M. Andersen, Karin Forsberg, Jochen H. Weishaupt, Seyed Babak Loghmani, Thorsten Grehl, Philip van Damme, Joachim Weis, Alberto Catanese

**Affiliations:** 1 Department of Neurology, University Hospital Ulm, 89081 Ulm, Germany; 2 Center for Rare Diseases (ZSE) Ulm, Ulm University Hospital Center for Rare Diseases, Ulm, 89081 Germany; 3 German Center for Neurodegenerative Diseases (DZNE), Ulm site, Ulm, 89081 Germany; 4 Institute of Anatomy and Cell Biology, Ulm University School of Medicine, Ulm, 89081 Germany; 5 Department of Neurosciences, Laboratory of Neurobiology and Leuven Brain Institute (LBI), KU Leuven-University of Leuven, Leuven, 3000, Belgium; 6 VIB, Center for Brain & Disease Research, Leuven, 3001 Belgium; 7 Institute of Neuropathology, RWTH Aachen University Hospital, Pauwelsstrasse 30, Aachen, 52074 Germany; 8 Biomedical Informatics, Data Mining and Data Analytics, University of Augsburg, Augsburg, 86159 Germany; 9 Institute of Molecular Oncology and Stem Cell Biology (IMOS), Ulm University Hospital, Ulm, 89081 Germany; 10 Brain Science Institute, Johns Hopkins University School of Medicine, Baltimore, MD USA; 11 Department of Neurology, Johns Hopkins University School of Medicine, Baltimore, MD USA; 12 Department of Neurology, Centre for ALS and Other Motor Neuron Disorders, Alfried Krupp Krankenhaus Rüttenscheid, Essen, 45131 Germany; 13 Institute of Human Genetics, Ulm University and Ulm University Medical Center, Ulm, Germany; 14 Core Facility Organoids, Ulm University, Ulm, 89081 Germany; 15 Department of Neurology, Brain Centre Rudolf Magnus, University Medical Centre Utrecht, Utrecht University, Utrecht, 3584 CG The Netherlands; 16 Department of Internal Medicine I, Division of Interdisciplinary Pancreatology, Ulm University Hospital, Ulm, 89081 Germany; 17 Medical Faculty, Unit for Single-Cell Genomics, Ulm University, Ulm, 89081 Germany; 18 Department of Clinical Sciences, Neurosciences, Umeå University , Umeå, Sweden; 19 Department of Neurology, University Hospitals Leuven, Leuven, 3000 Belgium; 20 Institute of Neuroanatomy, University Clinic RWTH Aachen, Wendlingweg 2, Aachen, 52074 Germany

**Correction:** **Acta Neuropathol Commun 14, 135 (2026)**

**https://doi.org/10.1186/s40478-026-02351-6**

In this article [1], the figure citations for Figs. 4 and 5, and 6 in the paragraph “Notably, while NEK1 kinase activity has been proposed. translocation in control MNs” were incorrect.

Notably, while NEK1 kinase activity has been proposed as the principal pathomechanism in NEK1-ALS [8], this hypothesis still required in vivo confirmation. NEK1 p.N598S MN cultures exhibited increased susceptibility to DNA damage and ciliary abnormalities (Fig. 4C-G), both processes known to depend on NEK1 kinase func­tion [9, 15]. This led us to hypothesize that the p.N598S variant might directly impact NEK1 kinase activity. To test this, we quantified the phosphorylation of the estab­lished direct NEK1 target YAP1 at tyrosine 407 (pTyr407) [16]. Both p.N598S- and p.R812*-mutant MN cultures showed reduced YAP1 pTyr407 levels compared with their isogenic counterpart (Fig. 5A), supporting reduced kinase activity. This was further assessed in whole lysates from MN cultures, and in immunopurified NEK1-WT and p.N598S. Overall kinase activity was markedly reduced in both p.N598S- and p.R812*-mutant cultures compared to wild-type MNs (Fig. 5B). Direct measure­ment of purified NEK1 confirmed that the p.N598S vari­ant displayed reduced kinase activity compared with wild-type NEK1 (Fig. 5C).

To further establish causality, we treated MNs derived from three healthy donors with the NEK1-specific inhibi­tor BSc5367. Exposure to 11.5 nM BSc5367 for 48 h sig­nificantly reduced kinase activity (Fig. 6A), decreased YAP1 phosphorylation at Tyr407 (Fig. 6B), and increased γH2A.X levels (Fig. 6C). Most notably, pharmacological inhibition of NEK1 was also sufficient to induce TDP- 43 nucleocytoplasmic translocation in control MNs (Fig. 6D).

These citations have now been corrected in the original publication.

The original article has been corrected.
