# Supplementary figures and images for: A rare missense variant impacting NEK1 kinase function is associated with ALS
Source: Acta Neuropathol Commun. 2026 Jun 25;14:135. doi: 10.1186/s40478-026-02351-6 (PMC13307424; doi:10.1186/s40478-026-02351-6)

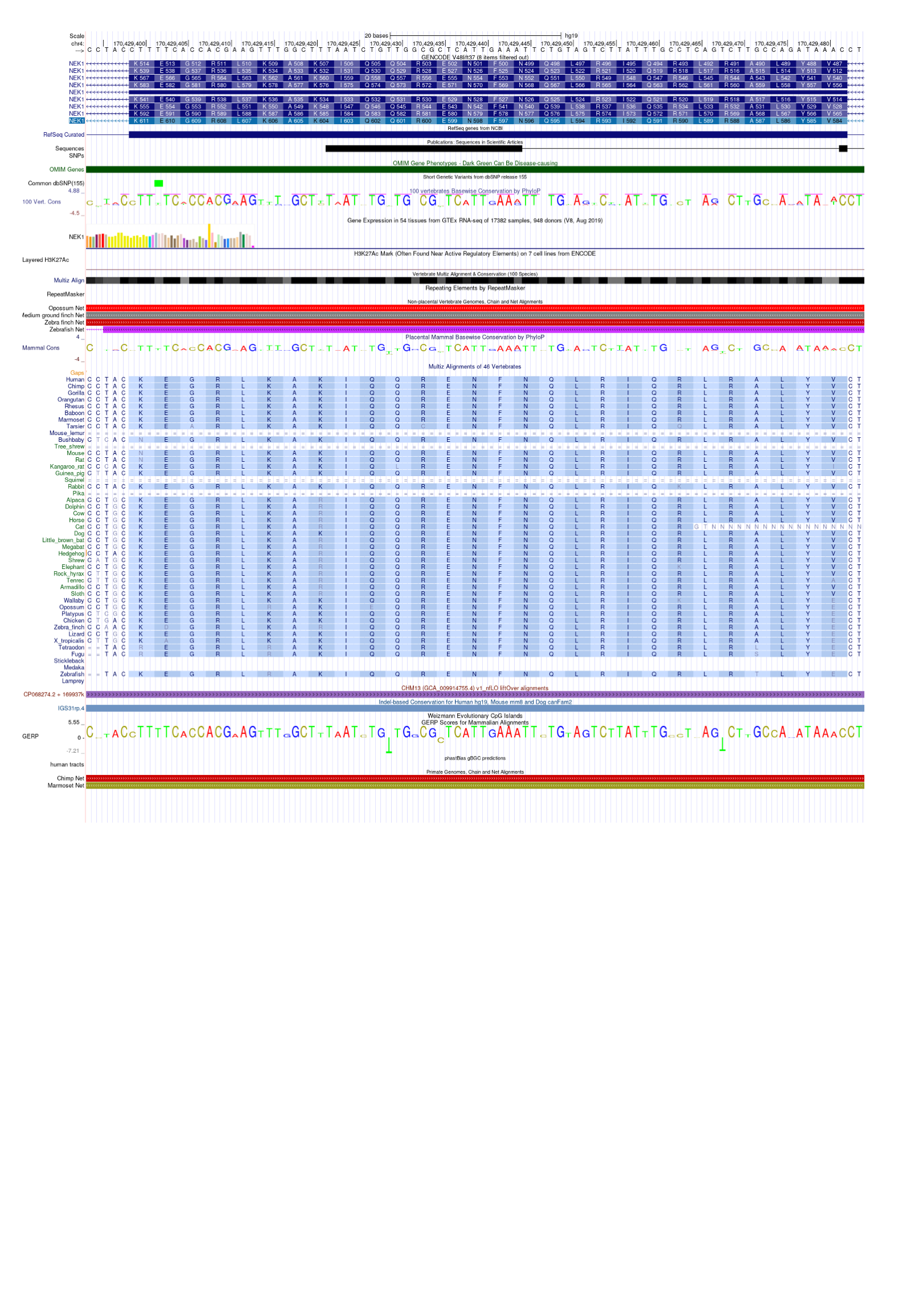

Supplement: Supplementary file 1 — Supplementary Material 1. Fig. 1 Conservation of NEK1 p.N598S variant across multiple species. [file 40478_2026_2351_MOESM1_ESM.tiff]

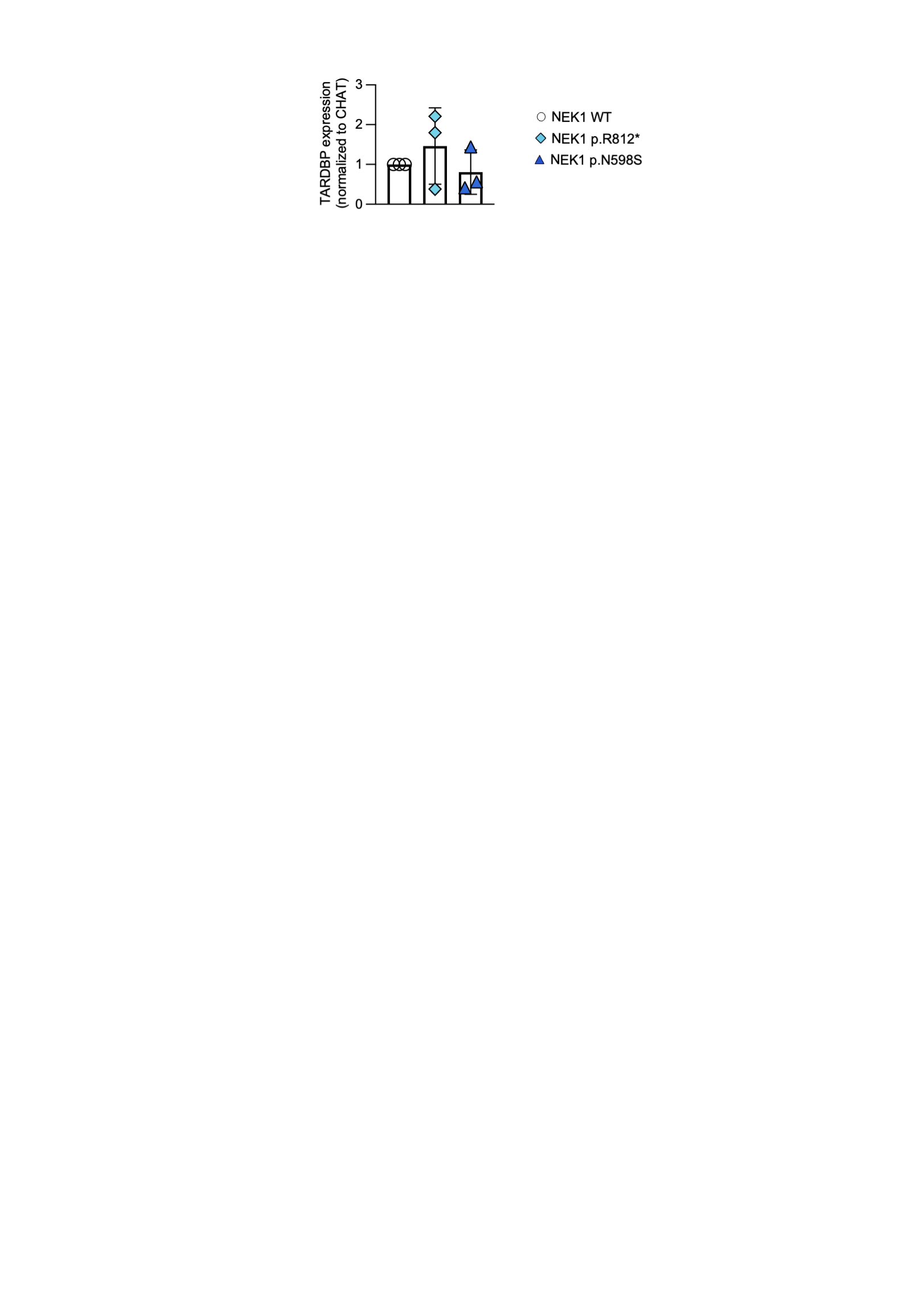

Supplement: Supplementary file 2 — Supplementary Material 2. Fig. 2 NEK1 mutations do not alter the expression levels of TARDBP in human motor neurons. qPCR analysis reveals comparable mRNA levels in NEK1-WT p.R812* and p.N598S cultures. N = 3 independent cultures for each genotype. [file 40478_2026_2351_MOESM2_ESM.tiff]

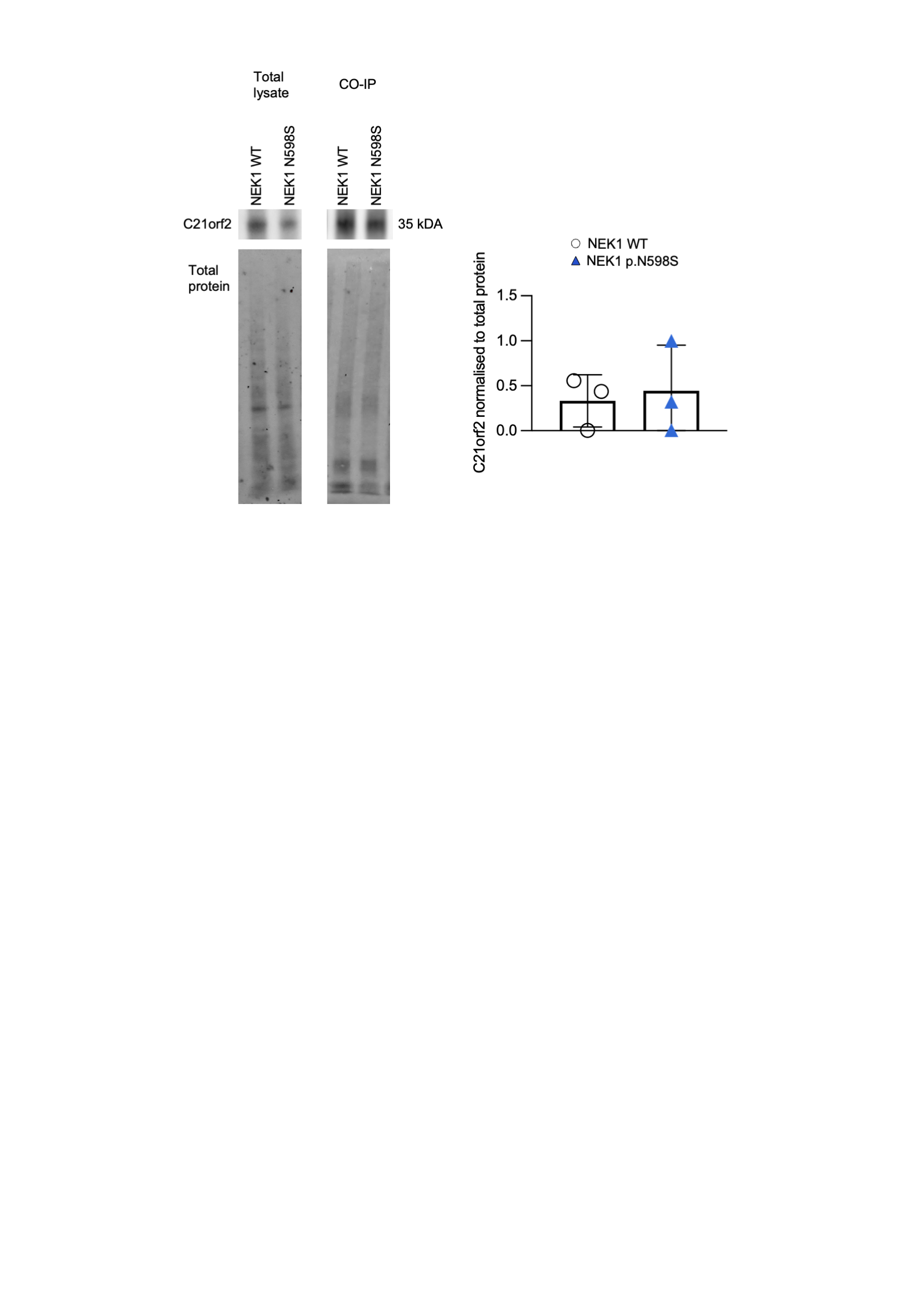

Supplement: Supplementary file 3 — Supplementary Material 3. Fig. 3 NEK1 p.N598S exacerbates TDP-43 pathology. Overexpression of the NEK1 p.N598S variant significantly increases the levels of cryptic STMN2 in HEK cells upon treatment with the proteasome inhibitor bortezomib. N = 3 independent cultures for each genotype. Mean ± SD of n = 3 independent differentiations; statistical significance was determined by two-way ANOVA with post hoc pairwise comparisons adjusted for multiple testing (FDR); p < 0.05 (*). [file 40478_2026_2351_MOESM3_ESM.tiff]

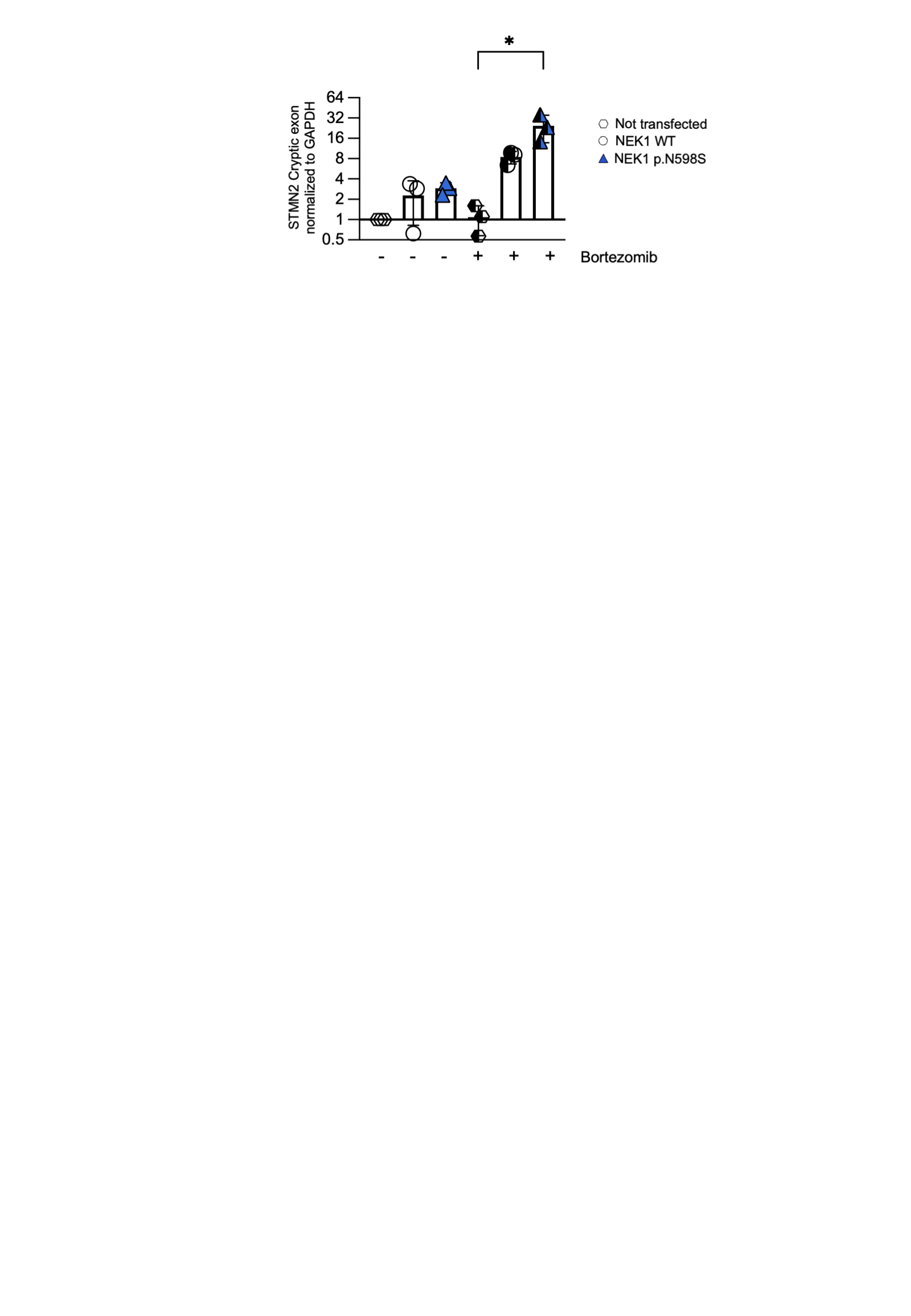

Supplement: Supplementary file 4 — Supplementary Material 4. Fig. 4 The p.N598S variant does not impact the interaction of NEK1 with C21orf2. Co-immunoprecipitation performed in HEK cells overexpressing either NEK1-WT or p.N598S. The levels of C21orf2 interacting with NEK1 are comparable in both groups.Mean ± SD of n = 3 independent experiments. [file 40478_2026_2351_MOESM4_ESM.tiff]
